# Synergistic Enhancement of Physicomechanical Performance and Microstructural Integrity in Hydrothermally Synthesized Autoclaved Lightweight Aggregates Through Quicklime–Fly Ash Blends

**DOI:** 10.3390/ma18122739

**Published:** 2025-06-11

**Authors:** Xue-Fei Chen, Xiu-Cheng Zhang, Ying Peng

**Affiliations:** 1 School of Civil Engineering, Putian University, Putian 351100, China; 2 Engineering Research Center of Disaster Prevention and Mitigation of Southeast Coastal Engineering Structures (JDGC03), Fujian Province University, Putian 351100, China

**Keywords:** fly ash, solid wastes, lightweight aggregates

## Abstract

Herein, fly ash aggregates (FAAs) were synthesized through a hydrothermal process, utilizing fly ash (FA) and quicklime at a temperature of 180 °C under saturated steam conditions. The study focused on analyzing the ramifications of varying quicklime content on the physicomechanical attributes of the resultant FAAs. A comprehensive examination of mineralogical composition, microstructure, insoluble matter content, and loss on ignition was conducted to elucidate the mechanisms through which quicklime influences the cylinder compressive strength of the FAAs. An observed trend indicated that as the quicklime content increased, the water requirement during the granulation process also increased. Consequently, there was a gradual augmentation in the water absorption capacity of the FAAs, accompanied by a progressive decrement in their apparent density. The augmentation in the filling effect, attributed to the hydration products, led to a steady rise in cylinder compressive strength as the quicklime content escalated from 5 wt.% to 25 wt.%. However, beyond this threshold, between 25 wt.% and 45 wt.%, a decrement in cylinder compressive strength was noted due to the deterioration of the micro-aggregate effect. The interplay between the filling effect and the micro-aggregate effect resulted in the cylinder compressive strength of the FAAs peaking at 13 MPa at a quicklime content of 25 wt.%. The overarching objective of this research is to propose an efficacious approach for mitigating solid waste, with a particular emphasis on reducing the burden of FA. This study provides insights into optimizing FAAs through the modulation of quicklime content, thereby fostering advancements in waste management and resource recovery.

## 1. Introduction

Fly ash (FA), a finely particulate byproduct of coal combustion, represents the principal solid waste generated by coal-fired power stations, particularly in regions such as Shanxi and Inner Mongolia in China, where substantial quantities of FA are produced annually [1]. Despite its inherent potential, the comprehensive utilization rate of FA hovers around 30%, leading to an annual accumulation that surpasses 100 million tons [2]. This colossal accumulation not only results in the squandering of valuable land resources but also exacerbates environmental pollution through the contamination of air, water, and soil. Consequently, promoting the resource-oriented utilization of FA is imperative for mitigating these detrimental effects and enhancing environmental quality [3]. Such initiatives are indispensable for fostering sustainable economic and social development in China and are fully aligned with the country’s dual carbon strategy development goals. Fly ash poses global environmental and industrial challenges yet holds significant resource recovery potential. Disposal in landfills risks soil/water contamination and airborne particulate hazards, particularly in developing nations facing land scarcity and inadequate regulations, while quality variability from diverse coal sources limits its use in high-performance concretes. Despite declining coal-fired power generation in regions like the U.S. and Europe, legacy stockpiles persist, even as emerging economies such as India and Poland mandate their use in infrastructure to reduce carbon footprints through supplementary cementitious applications. Innovative valorization pathways include geopolymer concretes replacing up to 80% of Portland cement, zeolite synthesis for water purification, and carbon capture integration via oxy-fuel combustion, with pilots in Canada and Australia demonstrating 15–20% CO₂ sequestration. Policy frameworks, including the EU’s Circular Economy Action Plan and the waste-to-resource strategies, drive recycling through incentives, though global disparities persist—Germany recycles 95% of its fly ash, whereas Sub-Saharan Africa lags below 10%. Cross-sector partnerships and pre-treatment technologies, such as triboelectrostatic beneficiation, are critical to scaling solutions amid coal phase-down pressures, ensuring this industrial byproduct transitions from a liability to a cornerstone of sustainable development. By harnessing advanced technologies and innovative approaches, it is possible to unlock the full potential of FA, transforming it from a waste product into a valuable resource that contributes to a green and sustainable future [4,5].

The utilization of FA for the production of lightweight aggregates (LWAs) emerges as a viable and promising strategy to address its accumulation and harness its potential [6,7,8]. LWAs, defined as materials exhibiting a bulk density below 1200 kg/m^3^ or an apparent density of less than 2000 kg/m^3^, possess a multitude of advantageous properties that render them highly suitable for diverse construction applications [9,10]. Their lightweight nature, coupled with high compressive strength, porous structure, and unique water absorption-storage-release characteristics, facilitates the creation of concrete with reduced density yet enhanced mechanical properties, including superior durability. Furthermore, LWAs exhibit a distinct ability to improve the thermal insulation and soundproofing capabilities of concrete [11]. These attributes not only enhance the efficiency of transportation and construction processes but also contribute to a reduction in carbon emissions throughout both the construction phase and the operational lifespan of the structure. As a result, LWAs have demonstrated considerable market potential and competitiveness in the realm of prefabricated buildings, high-rise structures, and long-span bridges.

In terms of classification, LWAs can be categorized into artificial and natural types based on their raw material sources. Given the finite and non-renewable nature of natural LWAs, the development of artificial LWAs has garnered significant attention. Artificial LWAs can be further subdivided into sintered and non-sintered types [12]. Traditional sintered LWAs, primarily utilizing clay as their raw material and undergoing high-temperature sintering processes at temperatures ranging from 1000 to 1200 °C or higher, are energy-intensive and pose challenges in terms of environmental sustainability, energy conservation, and emission reduction [13]. Conversely, non-sintered LWAs, such as autoclaved aggregates produced through hydrothermal synthesis utilizing calcium-rich and silica-rich raw materials, offer a more environmentally friendly and economically viable alternative for solid waste disposal [7,12,14]. These non-sintered LWAs not only exhibit excellent product performance but also contribute to the reduction in carbon emissions, conservation of natural resources, protection of the ecological environment, and enhancement of economic benefits.

FA is a widely utilized siliceous material in the production of autoclaved products, playing a crucial role in enhancing their structural integrity [15,16]. The fundamental chemical constituents of FA, viz. silicon dioxide (SiO_2_) and aluminum oxide (Al_2_O_3_), undergo reactions with calcium ions (Ca^2+^) in high-temperature and high-pressure aqueous environments. These reactions result in the formation of calcium silicate hydrate (C-S-H) [17,18,19] or calcium aluminate hydrate (C-A-H) phases, which are pivotal in imparting exceptional mechanical properties to autoclaved products. Despite these advantageous attributes, the application scope of these autoclaved products is predominantly confined to autoclaved bricks and autoclaved aerated concrete. Notably, research endeavors directed towards the development of autoclaved LWAs remain relatively scarce in the existing body of knowledge.

While some progress has been made in this regard, such as the successful preparation of autoclaved LWAs by Ma et al. [20], who employed epoxy propylene slag as the calcareous component and FA as the siliceous component, a comprehensive understanding of the variations in the physical and mechanical properties of autoclaved fly ash aggregates (FAAs) is still lacking. Moreover, the intricate mechanisms that govern these property variations have yet to be meticulously explored. A detailed investigation into these aspects is imperative for advancing the utilization of FAAs in various construction applications, thereby contributing to the broader field of sustainable building materials research.

To address this research gap, the novelty of this study is to undertake an in-depth exploration by utilizing FA as the siliceous raw material and selecting quicklime as the calcareous raw material. A hydrothermal synthesis process, coupled with autoclave curing, is employed to produce autoclaved FAAs. To gain a comprehensive understanding of the produced FAAs, a comprehensive battery of tests is conducted to measure their apparent density, cylinder compressive strength, water absorption capacity, mineral composition, microstructure, and loss on ignition. The primary objective of this investigation is to elucidate the intricate variations in the physical and mechanical properties of the autoclaved FAAs and to delve into the underlying mechanisms that govern these properties. This study thereby aims to contribute to the existing body of knowledge by providing theoretical foundations and technical support for the production of autoclaved FAAs. Moreover, this research is poised to extricate the path for the widespread adoption of FAAs across diverse construction applications, thereby facilitating the advancement of sustainable and environmentally benign building materials. Through this undertaking, it is envisioned that the utilization of FA will be further augmented, thereby aiding in the alleviation of environmental pollution, preservation of natural resources, and fostering sustainable progress.

## 2. Experimental Details

### 2.1. Materials

In the production of autoclaved fly ash aggregates (FAAs), fly ash (FA) and quicklime were selected as the fundamental raw materials. The FA originated from a power station located in Hefei, China. The ordinary Portland cement (OPC) of grade 52.5 was sourced from Jiangnan Onoda Cement Co., Ltd., also in Nanjing, China. The quicklime employed was acquired from Nanjing Changle Desiccant Co., Ltd., Nanjing, China, possessing an active calcium oxide (CaO) content of 90%, which is pivotal for the hydration reactions during the manufacturing process. Based on the XRF testing results, Table 1 delineates the comprehensive chemical composition of both the FA and the cement, facilitating a detailed understanding of their respective compositions.

### 2.2. Sample Preparation

**Figure 1:**
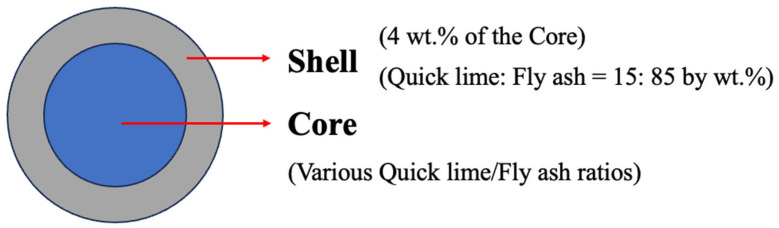
Schematic diagram of FAAs.

Autoclaved fly ash aggregates (FAAs) comprise two primary components: the shell and the core, as schematically depicted in Figure 1. Prior research has established a benchmark, indicating that the thickness of the shell constitutes approximately 4 wt.% of the core’s mass. This shell, which encapsulates the core, is formulated using a blend of FA and quicklime in a specific mass ratio of 85:15. Notably, all aggregates possess an identical shell composition, ensuring consistency across the experimental samples.

The core is composed of both quicklime and FA, with their combined mass summing up to 100%. To systematically investigate the impact of varying quicklime content on the physical and mechanical properties of autoclaved FAAs, quicklime was incrementally substituted for FA at equal intervals of 5% by mass. Among the various formulations tested, a reference group was established, consisting of 5 wt.% quicklime and 95 wt.% FA, denoted as F5F95. This reference group served as a point of comparison to evaluate the effects of altering the quicklime content.

To provide a detailed and comprehensive overview of the mix proportions employed in the production of FAAs, Table 2 presents a designed mix proportion scheme. This table encapsulates the specific quantities of FA and quicklime utilized in each formulation, thereby facilitating a thorough analysis of the impact of these variations on the resultant aggregate properties.

As delineated in Table 2, the quantities of quicklime and FA required for weighing are designated as *m*. These constituents are blended until a homogeneous dispersion is attained, whereupon water is introduced for digestion processing. The resultant amalgamation is subsequently encapsulated with plastic film and subjected to ambient temperature conditions for a minimum duration of 4 h to facilitate digestion, with the volume of water utilized for this purpose being noted as *m*_1_. Upon completion of digestion, the mixture is transferred to a mixer, where an adequate volume of water is incrementally introduced and thoroughly agitated until a tendency for powder agglomeration into fine granules emerges. The addition of water ceases once a wet powder mixture is obtained, at which point the quantity of mixing water utilized is documented as *m_2_*.

Subsequently, the wet powder mixture is deposited into a pelletizing disc and rotated to facilitate particle growth within the size range of 5–16 mm. Prior to rolling for a duration of 1–2 min, a dry powder mix corresponding to the shell composition is uniformly sprinkled over the surface to produce raw fly ash aggregates (FAAs). These raw FAAs are allowed to equilibrate indoors for a period of 24 h before being subjected to curing within a laboratory autoclave. The hydrothermal synthesis curing protocol employed in this experimental framework involves the gradual escalation of temperature from room level over 3 h to a curing temperature of 187 °C, maintained under a steam pressure of 1 MPa. This condition is sustained for 10 h before undergoing a controlled cool-down period spanning 2 h, returning to room temperature. Upon the conclusion of the curing process, the FAAs are extracted and dried to constant weight prior to the comprehensive evaluation of their physical and mechanical characteristics.

### 2.3. Testing

Physical properties of FAAs, including apparent density, water absorption, and cylinder compressive strength, were determined by GB/T 17431.2 [21], with calculations shown in Equations (1) to (4).

(1) $$\rho_{1}=\frac{10^{3}\times m}{V_{1}}$$

where $\rho_{1}$ is the loose bulk density (kg/m^3^); m is the dry mass of aggregates (g); $V_{1}$ is volume of the container (cm^3^).

The weighed dry FAAs (m) were soaked in water for 1 h to remove the influence of absorbing water of aggregates on apparent density, then treated to a saturated surface dry condition with wet towels. After that, the aggregates are poured into the graduated cylinder with adding an additional 500 cm^3^ of water, recording the final volume of the graduated cylinder (V). The apparent density of the aggregates was obtained according to the following Equation (2):

(2) $$\rho_{2}=\frac{10^{3}\times m}{V-500}$$

where $\rho_{2}$ is the apparent density (kg/m^3^); m is the dry mass of aggregates (g); V is volume of the final value of the graduated cylinder (cm^3^); 500 is the volume of additional water in the graduated cylinder (cm^3^).

Water absorption is the mass difference between the dry aggregates and water-saturated aggregates (immersed in water for 1 h and 24 h); 1 h water absorption and 24 h water absorption were calculated as Equation (3). Three parallel tests were conducted to generate the average value and the standard deviation.

(3) $$W_{i}=\frac{100\%\times \left(m_{i}-m\right)}{m}$$

where $W_{i}$ is the water absorption of aggregates (%), i = 1 is 1 h water absorption (%), i = 24 is the 24 h water absorption (%); m is the dry mass of aggregates (g); $m_{i}$ is the mass of aggregates after immersion in water (g), $m_{1}$ is the mass immersed in water for 1 h (g), and $m_{24}$ was the mass of aggregates immersed in water for 24 h (g).

Aggregates with diameters of 5–16 mm (70 wt.% 10~16 mm and 30 wt.% 5–10 mm) were put into a cylinder whose inner diameter is 56.9 mm and height is 120 mm, and then a load was applied uniformly on it. The value of pressure was recorded when the depth of punch indentation was 20 mm. This value shows the strength of aggregates, which is described as cylinder compressive strength, with a calculation as Equation (4).

(4) $$f=\frac{P}{A}$$

where f is the cylinder compressive strength (MPa); P is the recorded pressure when the depth of punch indentation was 20 mm (N); A is the area of a cylinder with an inner diameter of 56.9 mm and height of 120 mm (mm^2^).

The loss on ignition of FAAs is determined as per Equation (5).

(5) $$\mathrm{LOI}=\frac{m_{2}-m_{0}}{m_{1}}\times 100\%$$

where LOI is the loss on ignition of samples (%); $m_{0}$ is the mass of the crucible (g); $m_{1}$ is the mass of the sample before ignition(g); $m_{2}$ is the total mass of the sample and crucible after ignition (g).

The insoluble matter content of samples was tested as per Equation (6).

(6) $$R=\frac{100\%\times m_{2}}{m_{1}}$$

where R represents the insoluble matter content of the samples (in percentage), m₁ denotes the initial mass of the samples (in grams), and m₂ signifies the mass of the samples after acidification and calcination (in grams).

The particle size distribution of FA was analyzed by a laser diffraction particle analyzer (Model BT-9300S, Dandong Bettersize Instruments Ltd., Dandong, China). The crystalline phase of samples was characterized by X-ray diffraction (XRD, Model D8 Advance, Bruker, Germany) with CuKa radiation (k = 1.542 Å) at 40 kV. Microstructure was observed by a scanning electron microscope (SEM, Model Quant 250FEG, FEI, Eindhoven, The Netherlands). The surface of the samples was sputtered with a gold layer to improve the quality of the images.

## 3. Results and Discussion

### 3.1. Characterization of Raw Materials

**Figure 2:**
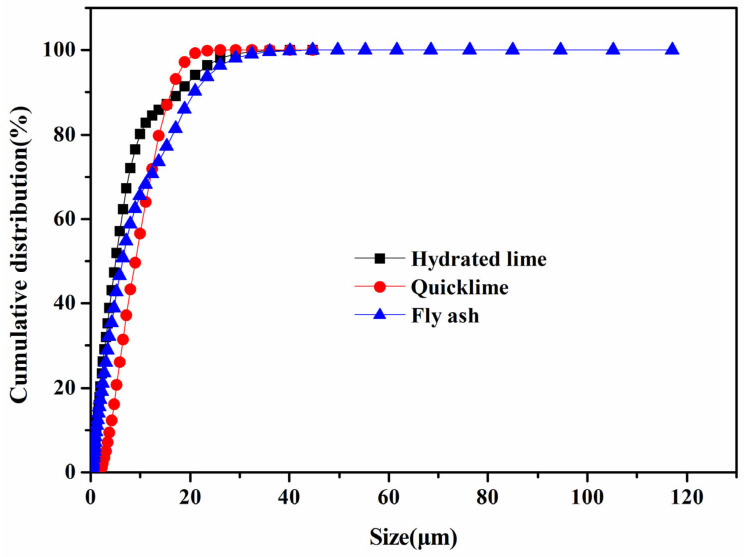
The particle sizes of quicklime, hydrated lime, and fly ash.

The granularity and specific surface area of raw materials exhibit a complex interplay with various process parameters, including the reaction kinetics, aqueous demands during the granulation procedure, the physicochemical attributes of aggregates, and the effectiveness of molding operations [22]. Finer raw materials are associated with elevated specific surface areas, a heightened abundance of interfacial contact points, more exhaustive chemical reactions, and augmented production of hydration byproducts. These factors collectively contribute to accelerated strength acquisition in autoclaved products. Furthermore, refined raw materials amplify molding efficiency and facilitate the manufacture of aggregates characterized by enhanced particle dimensions. In light of these considerations, a quantitative evaluation of the particle sizes of fly ash (FA), quicklime, and hydrated lime was conducted. The outcomes of this analysis are depicted in Figure 2.

Figure 2 elucidates that the particle size distribution of fly ash (FA) exhibits a broad spectrum, with notable peaks concentrated within the size ranges of 6–8 μm and 18–20 μm. The median diameter (D50), calculated as 6.38 μm, underscores the relatively diminutive size of FA particles, which facilitates the pelletization process. Conversely, quicklime demonstrates a peak concentration within the 12–14 μm range, with a median diameter (D50) of 9.01 μm. This observation hints at the capacity of fine quicklime particles to effectively disperse and form hydrated lime upon hydration. Hydrated lime, on the other hand, displays a diversified particle size distribution, marked by substantial concentration zones across four distinct intervals: 1–3 μm, 2–4 μm, 6–8 μm, and 20–22 μm. The median diameter (D50) for hydrated lime, approximated at 5.01 μm, indicates an enhanced pelletization capability post-hydration of quicklime.

The specific surface areas of fly ash (FA), quicklime, and hydrated lime have been quantified at 2.1599 m^2^/g, 1.5528 m^2^/g, and 28.5428 m^2^/g, respectively. Among these materials, hydrated lime exhibits the highest specific surface area, followed by FA and then quicklime. A correlation is evident where finer particle sizes are associated with larger specific surface areas, which aligns with the previously determined particle size sequence for these raw materials. During the granulation process, quicklime undergoes hydration to form hydrated lime. Consequently, the fly ash-based aggregates (FAAs) are composed primarily of hydrated lime and FA. Notably, hydrated lime possesses a larger specific surface area and smaller particle size compared to cement. When the proportion of quicklime is increased from 5 wt.% to 45 wt.% (in increments of 5 wt.%), the water consumption for the granulation of FAAs increases accordingly, ranging from a rise of 35.15% to 41.80%, 42.55%, 45.55%, 48.15%, 52.05%, 57.75%, 58.15%, and 68.40%, respectively. This significant increase in water consumption during the granulation process is poised to exert a substantial influence on the physical and mechanical properties of the resultant FAAs.

### 3.2. Physicomechanical Properties of Fly Ash Aggregates

#### 3.2.1. Water Absorption

**Figure 3:**
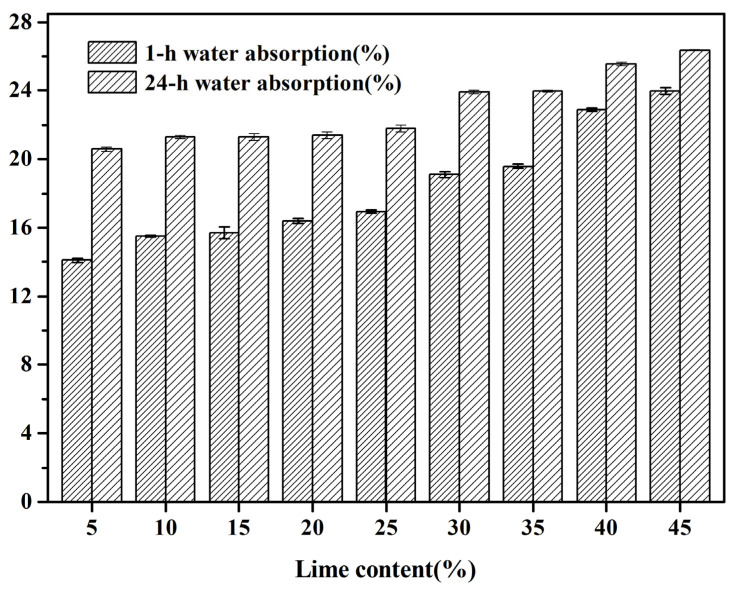
1 h water absorption and 24 h water absorption of FAAs L5F95-L45F55.

Figure 3 presents the water absorption characteristics of fly ash-based aggregates (FAAs). Upon examination, it is evident that with an escalating proportion of quicklime, there is a gradual increase in both the 1 h and 24 h water absorption of the FAAs. This observation suggests that a heightened content of quicklime leads to a decrement in the density of the FAAs and thereby augments their internal porosity, echoing previous studies [23,24]. During the autoclaving phase, the constituents Ca(OH)₂, SiO₂, and Al₂O₃ within the raw materials undergo hydrothermal synthesis reactions with water, giving rise to calcium silicate hydrate (C-S-H). This product serves to fill the voids or gaps within the material, exhibiting a notable filling effect. Furthermore, as the quantity of quicklime is incremented, there is a corresponding augmentation in the amount of hydration products, as corroborated by the X-ray diffraction (XRD) and scanning electron microscopy (SEM) analyses detailed below. From a theoretical standpoint, one would anticipate an enhancement in density and a subsequent decrement in water absorption due to this filling effect from hydration products [25].

However, contrary to this anticipation, the research reveals that for FAAs, an increase in the proportion of quicklime is accompanied by an elevation in their water absorption. This counterintuitive outcome arises from the elevated water consumption during the granulation process (as outlined in Table 2), leading to a heightened water-to-cement ratio. Consequently, during the curing phase, when moisture evaporates from the raw FAAs formed during granulation, voids are created due to initial defects, ultimately contributing to a decrease in density for these FAAs. This phenomenon, wherein increased initial defects are attributed to elevated water-to-cement ratios, is frequently observed in concrete as well. Furthermore, it underscores that while the formation of hydration products positively contributes through its filling effects on pores, the variations in granulation water volume exert a more pronounced influence on the water absorption of FAAs.

As illustrated in Figure 3, the water absorption of FAAs, specifically those ranging from L5F95 to L45F55, exhibits notably high values. The 1 h water absorption ranges from 14.1% to 23.98%, while the 24 h water absorption spans between 20.59% and 26.37%. The substantial water absorption capacity of these aggregates bestows significant internal curing effects upon cement matrices characterized by low water-to-cement ratios. This effect is instrumental in augmenting the strength of the interfacial transition zone, thereby leading to a marked enhancement in the mechanical properties of concrete. Furthermore, the utilization of these materials alleviates pertinent issues associated with drying shrinkage and autogenous shrinkage. Consequently, they demonstrate considerable potential as internal curing agents within high-performance concrete formulations characterized by low water-to-binder ratios. The incorporation of these aggregates can contribute to the development of concrete with enhanced durability and mechanical performance, making them suitable for applications requiring superior structural integrity.

#### 3.2.2. Apparent Density

**Figure 4:**
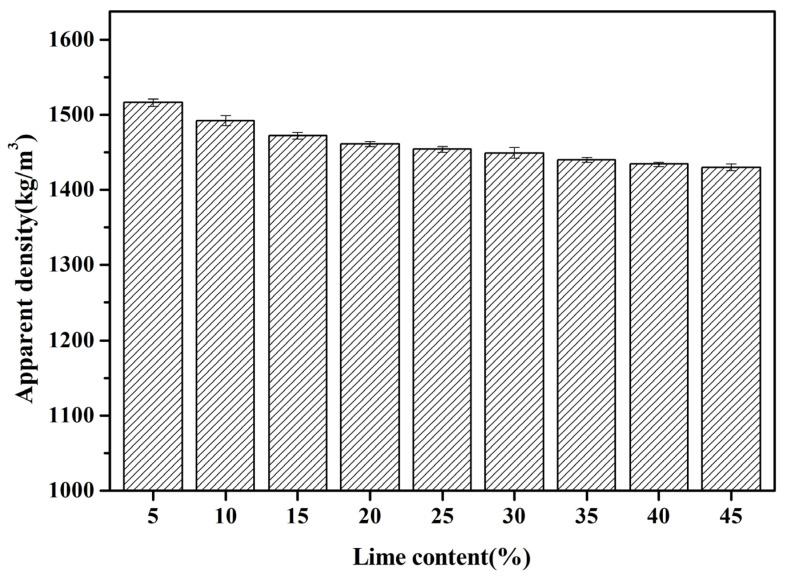
Apparent density of FAAs L5F95-L45F55.

Figure 4 exhibits the apparent density variations of fly ash aggregates (FAAs) as a function of increasing quicklime content, ranging from 5 wt.% to 45 wt.% in increments of 5 wt.%. As the quicklime concentration escalates, the density of the FAAs diminishes progressively from 1516 kg/m^3^ to 1430 kg/m^3^. This decrement in apparent density can be rationalized by the hydration process of quicklime into hydrated lime. Consequently, a cubic meter of the aggregate comprises hydrated lime and fly ash, where hydrated lime possesses an apparent density of approximately 2240 kg/m^3^, which is inferior to that of FA, estimated at 2450 kg/m^3^. Thus, as the proportion of quicklime augments, the overall apparent density of the FAAs exhibits a gradual decline.

Additionally, Figure 3 illustrates a notable decrease in the water absorption capacity of the FAAs with an increment in quicklime content. This observation implies a decrement in the compactness of the aggregates, which is another pivotal factor contributing to the reduction in apparent density for the FAAs, particularly from L5F95 to L45F55, due to the higher incorporation of quicklime. The apparent density of the FAAs amended with quicklime, as depicted in Figure 4, spans from 1430 kg/m^3^ to 1516 kg/m^3^. In accordance with the specifications stipulated in GB/T17431.1 [26], particles with an apparent density below 2000 kg/m^3^ are categorized as lightweight aggregates (LWAs). Hence, the apparent densities of all FAAs, ranging from L5F95 to L45F55, fall below 2000 kg/m^3^, thereby fulfilling the criteria for lightweight materials.

#### 3.2.3. Cylinder Compressive Strength

**Figure 5:**
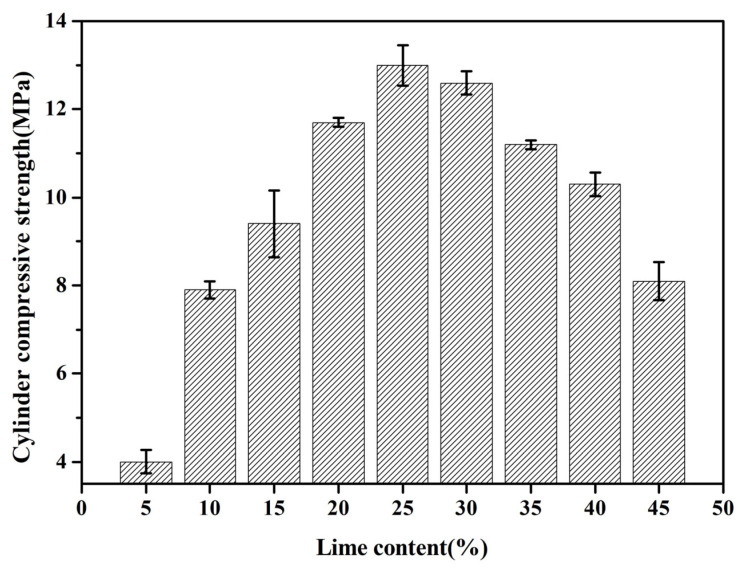
Cylinder compressive strength of FAAs L5F95–L45F5.

Figure 5 illustrates the compressive strength of cylindrical specimens fabricated using fly ash aggregates. The compressive strength of these specimens demonstrates a biphasic trend, initially augmenting and subsequently diminishing with an escalating quicklime content. Precisely, as the quicklime content ascends from 5 wt.% to 25 wt.%, the compressive strength of the cylindrical specimens augments from 4 MPa to 13 MPa. However, upon further increasing the quicklime content from 25 wt.% to 45 wt.%, the compressive strength decreases, diminishing from 13 MPa to 8.1 MPa. Notably, at a lime content of 25 wt.%, the FAA composition denoted as L25F75 attains its peak compressive strength. This observation can be attributed to the presence of an optimal calcium oxide to silicon dioxide (CaO/SiO_2_) ratio within L25F75. A CaO/SiO_2_ ratio that is too low leads to inadequate hydration products filling the interstitial spaces between fly ash particles, resulting in an abundance of defects and, consequently, a diminished compressive strength [27,28,29]. Conversely, an excessively high CaO/SiO_2_ ratio tends to produce surplus calcium hydroxide (Ca(OH)_2_) under autoclaved curing conditions, which retards the formation of high-strength, low-alkali calcium silicate hydrates. Ultimately, this results in a decrease in the compressive strength of the FAAs.

In comparison to L5F95, the cylinder compressive strength of FAAs L10F90, L15F85, L20F80, L25F75, L30F70, L35F65, L40F60, and L45F55 exhibited increments of approximately 97.5%, 135%, 192.5%, 225%, 215%, 180%, 157.5%, and 102.5%, respectively. This underscores a substantial augmentation in the cylinder compressive strength of FAAs with an elevated quicklime dosage. Notably, the enhancement in cylinder compressive strength peaked at an impressive 225% when the quicklime content attained 25 wt.%. Prior research has established that, with a consistent cement mortar, the mechanical properties of lightweight aggregates (LWAs) are pivotal in determining the overall strength of concrete [30,31,32]. Consequently, LWAs possessing superior strength correspond to higher concrete strength. Hence, the enhancement of FAAs’ strength through an increased quicklime content holds profound implications for bolstering the strength of lightweight aggregate concrete, suggesting a potential pathway for optimizing the mechanical performance of such materials.

The quicklime industry is recognized as a sector with significant environmental pollution, with the production of 1 ton of quicklime generating approximately 0.785 tons of carbon dioxide (CO_2_) emissions. Figure 5 presents the cylinder compressive strength results for FAAs with various quicklime proportions, specifically L30F70, L35F65, L40F60, and L45F55, containing 30 wt.%, 35 wt.%, 40 wt.%, and 45 wt.% quicklime content, respectively. While these proportions demonstrate positive outcomes, their strengths are still inferior to those achieved by FAA L25F75 with only 25 wt.% quicklime content. The utilization of higher quicklime proportions in FAAs not only fails to achieve optimal mechanical performance but also exacerbates carbon emissions, posing substantial challenges to the sustainable development of the aggregate industry. Upon considering both carbon emissions and mechanical properties, it is concluded that the proportion of quicklime utilized in the preparation of FAAs should not surpass 25 wt.%.

The cylinder compressive strength of FAAs, ranging from L5F95 to L45F55, spans from 4 MPa to 13 MPa, as illustrated in Figure 5. According to the specifications stipulated in GB/T 17431.1, lightweight aggregates (LWAs) with a cylinder compressive strength exceeding 6.5 MPa are categorized as high-strength LWAs. Notably, except for L5F95, all other FAAs demonstrate a cylinder compressive strength greater than 6.5 MPa. Consequently, FAAs L10F90 to L45F55 can be classified as high-strength LWAs, highlighting their potential for use in applications requiring superior mechanical properties.

### 3.3. Micro-Structure Analysis

#### 3.3.1. XRD Analysis

**Figure 6:**
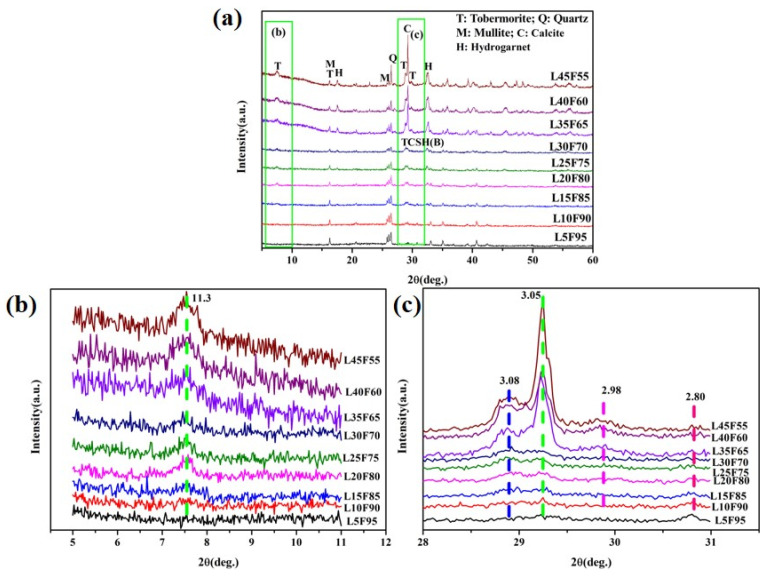
XRD patterns of FAAs L5F95–L45F55. (**a**) mullite and quartz; (**b**) tobermorite; (**c**) CSH(B).

The X-ray diffraction (XRD) patterns of the fly ash-based aggregates (FAAs) are depicted in Figure 6. Figure 6a reveals that the phase composition of the FAAs comprises residual mullite (3Al_2_O_3_·2SiO_2_) and quartz. Figure 6c exhibits diffuse diffraction peaks for the samples L5F95 to L45F55 at 2θ = 30.801° and 2θ = 29.246°, indicating the presence of poorly crystalline hydration phases, specifically amorphous calcium silicate hydrate (CSH(B)), characterized by peak values at 2.80 Å and 3.05 Å, respectively.

In Figure 6b, when the quicklime content increases to 10 wt.%, the FAAs L10F90 exhibit the presence of tobermorite, with characteristic peak values at 11.3 Å, 3.08 Å, and 2.98 Å. Furthermore, as illustrated in Figure 6a, an additional increase in quicklime content by 15 wt.% results in the emergence of a new hydration product—hydrogarnet (3CaO·Al_2_O_3_·SiO_2_·4H_2_O)—in sample L15F85, with main characteristic peaks at approximately 5.05 Å and 2.75 Å.

Figure 6c indicates that when the quicklime content ranges from 35 wt.% to 45 wt.%, the FAAs C35F65, C40F60, and C45F55 exhibit calcite formation at a diffraction angle of approximately 2θ = 29.246°. This phenomenon may be attributed to carbonation resulting from the higher amounts of quicklime. However, despite this observation, the broadening of diffraction peaks still suggests the coexistence of CSH(B). In summary, the primary phases present in the FAA L5F95 consist mainly of the hydrated product CSH(B), along with residual mullite and quartz. Regarding the FAAs L10F90, they include hydrated product CSH(B), tobermorite, as well as residual mullite and quartz. Meanwhile, the FAAs L15F85 to L45F55 predominantly contain hydrated products such as CSH(B), tobermorite, hydrogarnet, alongside residual mullite and quartz. This analysis elucidates that increasing amounts of quicklime significantly influence both the types and varieties of hydration products formed during processing, particularly evident when higher proportions are utilized.

Furthermore, the SiO_2_ content within fly ash is 53.47 wt.%, whereas the CaO content stands at 2.98 wt.%. The effective CaO content in quicklime amounts to 90 wt.%. When the quicklime contents are adjusted to 30 wt.%, 35 wt.%, 40 wt.%, and 45 wt.%, the CaO/SiO_2_ ratios of the raw materials for the FAAs L30F70, L35F65, L40F60, and L45F55 can be calculated as 0.83, 1.02, 1.26, and 1.53, respectively, based on their respective chemical compositions. Despite these ratios approaching or exceeding 0.83, Figure 6a still reveals the persistence of quartz diffraction peaks in the FAAs C30F70 to C45F55. This observation implies an incomplete reaction of quartz and suggests an excess of Ca^2+^ ions, stemming from an overly high lime addition. Therefore, the inclusion of quicklime at dosages ranging from 30 wt.% to 45 wt.% in the preparation of FAAs remains excessive.

As the quantity of quicklime increases, the intensity of the diffraction peaks corresponding to mullite and quartz decreases (Figure 6a), while the diffraction peak for CSH(B) gradually broadens (Figure 6c). This signifies that an elevated calcium source content facilitates the formation of CSH(B), which subsequently reacts with silicate ions in solution to produce tobermorite. Consequently, tobermorite emerges in the FAA L10F90, and its diffraction peak intensity augments with higher quicklime content (Figure 6b,c). Under conditions where Ca^2^⁺ ions are abundant, due to the excessive presence of Al₂O₃ in FA, hydrogarnet appears in L25F75. Furthermore, as the quicklime content escalates, the intensity of the hydrogarnet diffraction peaks intensifies (Figure 6a). The aforementioned phenomena underscore that the incorporation level of quicklime markedly influences both the quantity of hydration products and the contents of mullite and quartz. Elevating the quicklime dosage can augment the amount of hydration products formed.

#### 3.3.2. Insoluble Matter and Loss on Ignition

**Figure 7:**
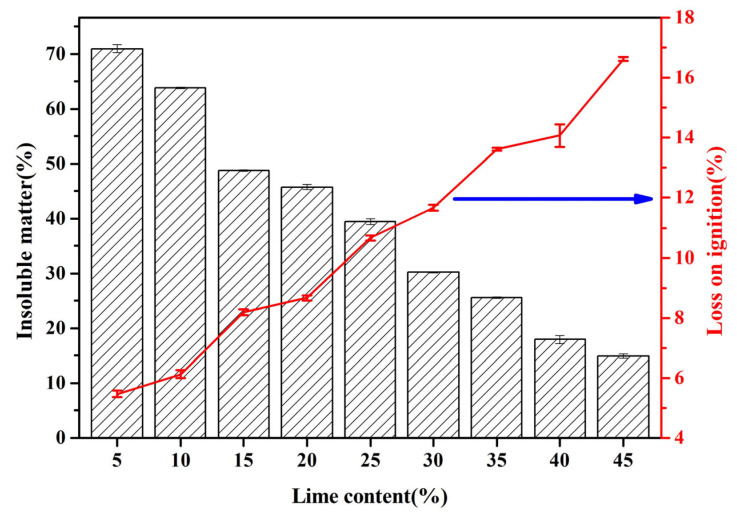
Insoluble matter and loss on ignition of FAAs L5F95~L45F55.

Figure 7 presents the insoluble residue and loss on ignition (LOI) in FAAs. As the proportion of quicklime is incremented from 5 weight percent (wt.%) to successive levels of 10 wt.%, 15 wt.%, 20 wt.%, 25 wt.%, 30 wt.%, 35 wt.%, and ultimately reaching 40 wt.% and 45 wt.%, a progressive diminution is observed in the insoluble matter content within the FAAs, decreasing from 70.94% to 63.83%, 48.71%, 45.72%, 39.48%, 30.22%, 25.57%, and finally, to 14.96%. This decrement signifies a reduction in the residual quartz and mullite contents within the FAAs, which corroborates the diminishing intensity of X-ray diffraction (XRD) peaks corresponding to quartz and mullite, as illustrated in Figure 6a, with the augmentation of quicklime content. Quartz and mullite act as structural scaffolds in FAAs, exhibiting a micro-aggregative effect. Consequently, the reduction in insoluble matter results in a decline in the cylinder compressive strength when the quicklime content rises from 25 wt.% to 45 wt.% (Figure 5). Nevertheless, it is noteworthy that despite the discernible downward trend in insoluble matter content (Figure 6), an augmentation in cylinder compressive strength is observed when the quicklime content increases from 5 wt.% to 25 wt.% (Figure 5). Further elucidation of this phenomenon necessitates additional analysis utilizing scanning electron microscopy (SEM).

The SiO_2_ content in FA is 53.47 wt.%, whereas the CaO content is 2.98 wt.%. The effective CaO content in the quicklime utilized is 90 wt.%. When varying amounts of quicklime are added at levels of 5 wt.%, 10 wt.%, 15 wt.%, 20 wt.%, 25 wt.%, 30 wt.%, 35 wt.%, 40 wt.%, and 45 wt.%, the corresponding CaO/SiO_2_ ratio of the raw materials in FAAs can be computed as follows: 0.15, 0.26, 0.38, 0.51, 0.66, 0.83, 1.03, 1.26, and 1.53, respectively. Based on the quantified insoluble residue content, the corresponding CaO/SiO_2_ ratios for the hydration products are calculated to be 0.53, 0.72, 0.74, 0.94, 1.09, 1.19, 1.38, 1.54, and 1.80, respectively. A detailed presentation of the calculation results is provided in Table 3.

The CaO/SiO_2_ ratio of the hydration products in the FAAs L5F95, L10F90, and L15F85 is consistently below 0.83. Taking L5F95 as an illustrative example, calculations presented in Table 3 reveal that the CaO/SiO_2_ ratio of its hydration products is 0.53. This suggests that FAA L5F95 must necessarily contain hydration products with a CaO/SiO_2_ ratio exceeding 0.53. The existence of hydration products with a CaO/SiO_2_ ratio greater than 0.53, specifically calcium silicate hydrates of type B (CSH(B), represented as 0.8–1.5 CaO·SiO_2_·nH_2_O), is corroborated by the X-ray diffraction (XRD) pattern depicted in Figure 6. When the quicklime content varies from 25 wt.% to 45 wt.%, the CaO/SiO_2_ ratio of the hydration products ranges between 0.94 and 1.80. Notably, in the case of the fly ash ceramic aggregate L45F55, this ratio attains its maximum value of 1.80. Using L45F55 as an exemplar indicates that its hydration products should comprise both those with calcium-to-silicon ratios above and below 1.80 concurrently. Tobermorite, characterized by a calcium-to-silicon ratio of approximately 0.83, which is lower than that of CSH(B), falls within this range. Consequently, it can be inferred that when the quicklime content is at or near 45%, the fly ash ceramic aggregate C45F55 must contain both tobermorite and CSH(B), as further substantiated by the XRD pattern presented in Figure 6. In addition to these constituents, it is imperative that the FAAs also encompass hydration products with a CaO/SiO_2_ ratio exceeding 1.80. To confirm the presence of such products, further analysis utilizing scanning electron microscopy coupled with energy-dispersive X-ray spectroscopy (SEM-EDS) techniques will be required.

The results concerning the loss on ignition (LOI) content in FAAs are illustrated in Figure 7. As the concentration of quicklime progressively increases from 5 wt.% to 10 wt.%, 15 wt.%, 20 wt.%, 25 wt.%, 30 wt.%, 35 wt.%, and ultimately attains 40 wt.% and 45 wt.%, the LOI content of the FAAs escalates from 5.47% to 6.13%, 8.19%, 8.67%, 10.66%, 11.67%, 13.62%, and finally reaches 16.62%. This increment signifies that the content of hydration products in FAAs augments with higher quantities of quicklime. This observation is congruent with the trend depicted in Figure 6, wherein the intensity of diffraction peaks corresponding to tobermorite and other hydrated phases in the X-ray diffraction (XRD) patterns intensifies as the quicklime dosage increases. Furthermore, the enhanced content of hydration products, leading to improved filling effects within the material structure, results in a discernible rise in the cylinder compressive strength. This increase in compressive strength is evident when the quicklime dosage is elevated from 5 wt.% to 25 wt.%, as illustrated in Figure 5.

#### 3.3.3. SEM Analysis

**Figure 8:**
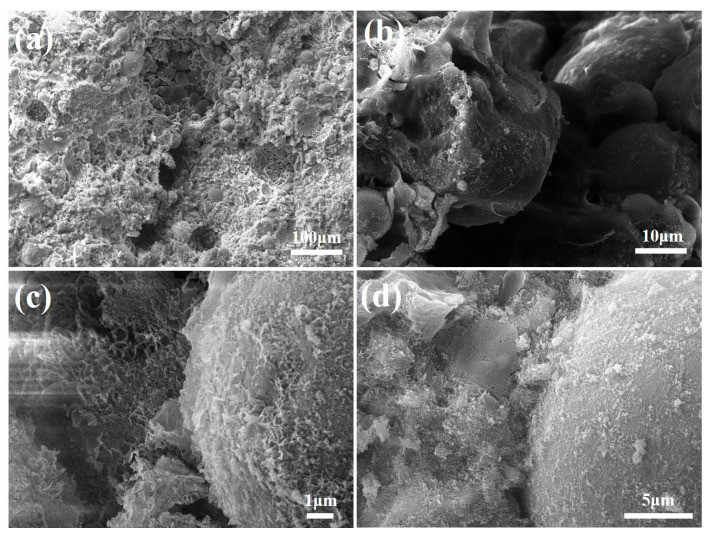
SEM images of FAA L5F95. (**a**) loose microstructure within the FAA matrix (large scale); (**b**) loose microstructure within the FAA matrix (small scale); (**c**) flaky hydration products (small scale); (**d**) flaky hydration products (large scale).

The SEM micrographs of the FAA specimen L5F95 are presented in Figure 8. Upon examination of Figure 8a,b, it is evident that voids are interspersed among the spherical FA particles, indicative of a relatively loose microstructure within the FAA matrix. This observation implies that the generation of hydration products is limited, leading to weak interconnections between the spherical FA particles and a preponderance of pores. Despite the pronounced micro-aggregate effect exhibited by these unreacted FA particles, their presence contributes to the attainment of the lowest cylinder compressive strength by L5F95, as illustrated in Figure 5. In contrast, Figure 8c,d reveal the development of flaky hydration products, with lengths approximating 1 to 2 μm, on both the surfaces of the spherical FA particles and within the surrounding matrix. These hydration products form a network structure through mutual interlocking, enhancing the overall coherence and integrity of the FAA matrix.

**Figure 9:**
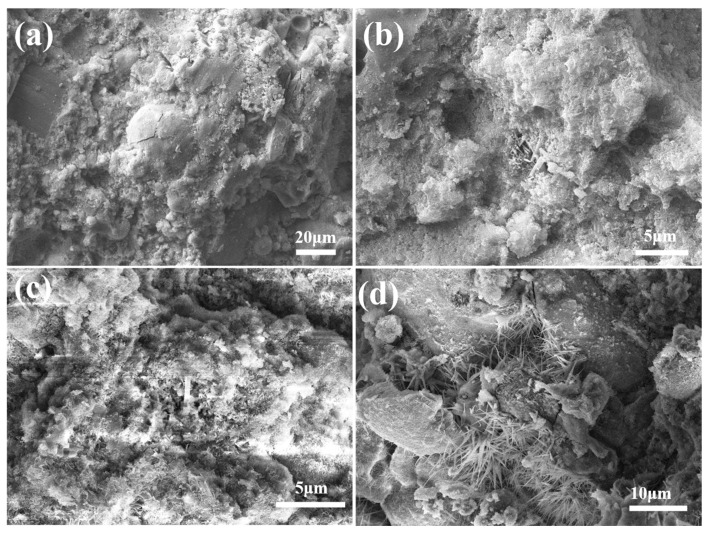
SEM images of FAA L25F75. (**a**) spherical fly ash (large scale); (**b**) spherical fly ash (small scale); (**c**) needle-like tobermorite (small scale); (**d**) needle-like tobermorite (large scale).

The SEM images of the FAA specimen L25F75 are presented in Figure 9. Upon inspection of Figure 9a,b, it becomes evident that the identification of spherical fly ash (FA) particles is challenging, with only a limited number discernible in Figure 9a. Furthermore, the surfaces of these spherical particles are enveloped by a substantial layer of hydration products. The interstitial spaces between the spherical FA particles are replete with hydration products, which effectively bond the unreacted FA particles together, thereby forming a cohesive and integrated structure. Additionally, the micro-aggregate effect exerted by unreacted mullite and quartz (as observed in Figure 6) contributes significantly to the attainment of the highest cylindrical compressive strength exhibited by the FAA specimen L25F75. In Figure 9c,d, needle-like tobermorite phases are observed to interpenetrate within a fibrous matrix composed of hydrated calcium silicate. This interlocking structure serves as a primary contributor to the mechanical strength of the FAA specimen, enhancing its overall load-bearing capacity.

**Figure 10:**
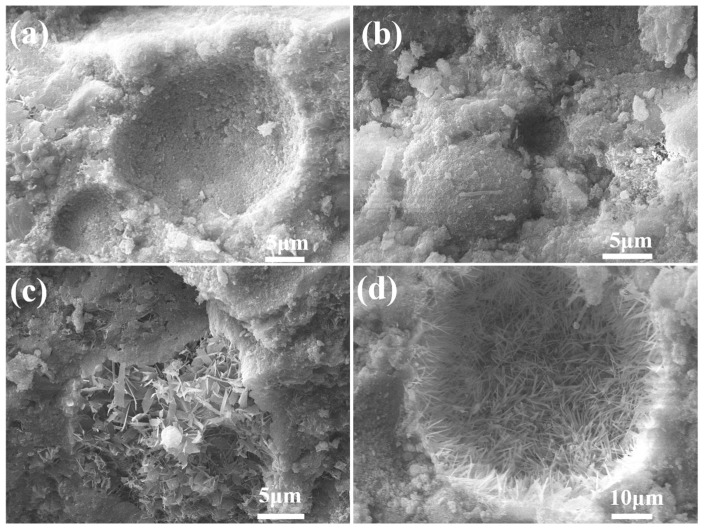
SEM images of FAA L35F65. (**a**) spherical fly ash; (**b**) dense, cotton-like layer of hydration products; (**c**) fibrous tobermorite (small scale); (**d**) fibrous tobermorite (large scale).

The SEM images of the FAA specimen L35F65 are presented in Figure 10. In Figure 10a, the spherical fly ash (FA) particles are not readily discernible, indicating a high degree of embedding or alteration. In Figure 10b, a dense, cotton-like layer of hydration products envelops the surface of the spherical FA particles, fostering a tight coupling between the particles and the surrounding matrix. Figure 10c,d reveal the presence of pores, approximately 10 μm in diameter, that are filled with needle-like tobermorite crystals. These crystals interconnect to form a cohesive and integrated structure, which constitutes one of the primary contributors to the mechanical strength of the FAA specimen. The network architecture formed by the interlocking tobermorite crystals is crucial for enhancing the load-bearing capacity of the material. Furthermore, in Figure 10c,d, it is evident that fibrous tobermorite fills the pores, demonstrating the efficacy of high-temperature and high-pressure curing conditions in promoting the formation and distribution of hydration products. These conditions facilitate the effective infiltration of hydration products into internal pores and other defects within the ceramic particles, thereby mitigating their loose porosity and enhancing the overall density and structural integrity of the FAA specimen.

**Figure 11:**
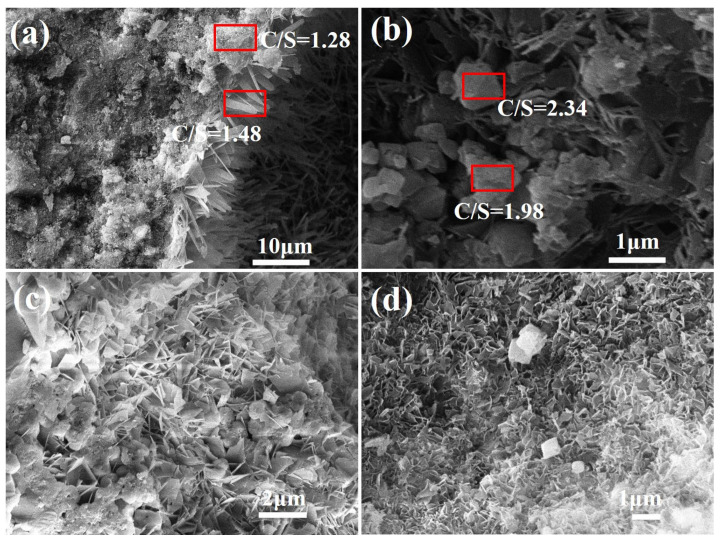
SEM images of FAA L45F55. (**a**) flaky hydration products (large scale); (**b**) CaO/SiO_2_ ratio; (**c**) flaky hydration products (middle scale); (**d**) flaky hydration products (small scale).

The microstructure of the FAA L45F55 was observed, with the results presented in Figure 11. In Figure 11a,c,d, distinct flaky hydration products are clearly visible, which interconnect to form a network structure. An EDS analysis of the flaky hydration products is shown in Figure 11a. It can be noted that the CaO/SiO_2_ ratio of these flaky hydration products is 1.28 and 1.48. Based on XRD results, it can be confirmed that this hydrated product corresponds to CSH(B).

Additionally, it was observed in Figure 11b that fibrous hydration products were present within the FAAs. EDS analysis revealed that the CaO/SiO_2_ ratios of these hydration products were approximately 1.98 and 2.34, indicating a high-alkalinity, low-strength hydrated calcium silicate. Assuming that all Ca^2^⁺ ions in the FAAs L45F55 participated in the hydrothermal synthesis reaction, we can calculate the CaO/SiO_2_ ratio of the hydration products in the FAAs to be around 1.80 based on its insoluble matter content (Figure 7, 14.96 wt.%). Therefore, it is inferred that FAA L45F55 contains hydration products with a CaO/SiO_2_ ratio exceeding 1.80. This finding supports the results obtained from insoluble matter content analysis as verified by SEM-EDS. Furthermore, it should be noted that hydrated calcium silicates with a high CaO/SiO_2_ ratio exhibit lower strength compared to tobermorite. Thus, their presence constitutes another significant factor contributing to the reduced strength of FAA L45F55.

**Figure 12:**
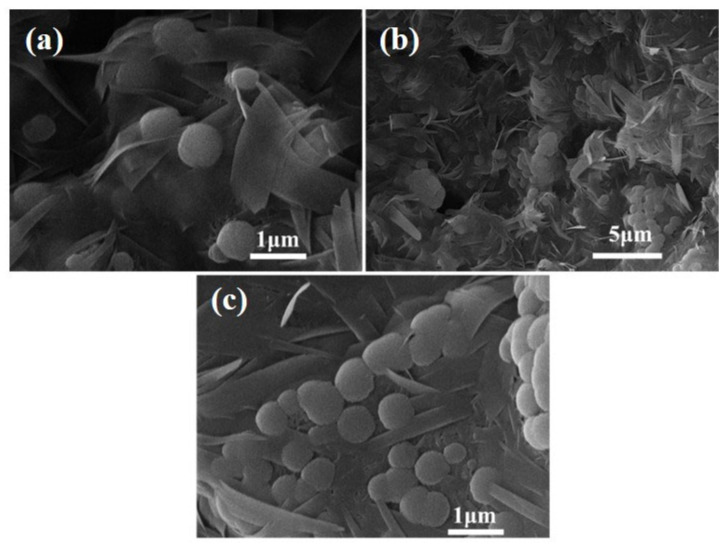
SEM images of FAA L45F55. (**a**) hydrated silica phase; (**b**) coarse-grain size octahedral material; (**c**) hydrogarnet.

In Figure 12, concerning the FAA L45F55, a new crystal was identified within the hydrated silica phase. This crystal exhibits coarse-grain size and high crystallinity, taking on an octahedral form and existing in a granular state. Correlating with the XRD analysis results presented in Figure 6, the newly observed crystal is identified as hydrogarnet.

#### 3.3.4. The Mechanism of Strength Development in FAAs

The aforementioned research reveals a trend wherein the cylinder compressive strength of FAAs exhibits an initial increase followed by a decrease, attaining its maximum value at a quicklime content of 25 wt.%. Based on an in-depth analysis of the insoluble matter content, mineral composition, and microstructure depicted in Figure 6, Figure 7, Figure 8, Figure 9, Figure 10, Figure 11 and Figure 12, in conjunction with the cylinder compressive strength data presented in Figure 4, it can be deduced that at a quicklime content of 5 wt.%, a pronounced micro-aggregate effect is present within the FAAs. Nonetheless, the absence of adequate hydration product filling effects results in significant porosity between the FA particles, leading to a loose microstructure that precludes the attainment of optimal cylinder compressive strength. This observation underscores the inadequacy of the micro-aggregate effect alone in ensuring optimal mechanical performance.

In the range where the quicklime content varies from 30 wt.% to 45 wt.%, although the hydration products provide sufficient filling effects, the diminution of the micro-aggregate effect contributes to a decline in cylinder compressive strength. This finding indicates that even with the presence of hydration product filling effects, they are insufficient to elevate the cylinder compressive strength of FAAs to an optimal level in isolation. At a quicklime content of 25 wt.%, the cylinder compressive strength reaches its zenith at 13 MPa, underpinned by the synergistic interplay of both the micro-aggregate effect and enhanced hydration product filling effects. This outcome demonstrates that the attainment of optimal cylinder compressive strength values in FAAs necessitates the coexistence and synergy of these two phenomena. Consequently, this study posits a collaborative effect between the micro-aggregate and filling effects, emphasizing that optimal cylinder compressive strengths can only be achieved through their concerted action. The elucidation of this synergistic effect provides deeper insights into the mechanisms underlying the formation of superior mechanical properties in FAAs under conditions of optimal lime incorporation. This understanding is pivotal for the development and optimization of the material with enhanced performance characteristics.

## 4. Conclusions

The raw materials utilized in this investigation consist of quicklime and fly ash (FA). Through the application of a low-energy hydrothermal synthesis method, augmented by steam curing, we were able to synthesize fly ash aggregates (FAAs) via calcium-silicate reactions. The outcomes of this study reveal the following insights:

(1) As the quicklime content increases from 5 wt.% to 25 wt.%, the enhancement in the filling effect of hydration products contributes to a gradual elevation in the cylinder compressive strength of the FAAs. However, beyond this threshold, specifically within the range of 25 wt.% to 45 wt.% quicklime content, a decrement in cylinder compressive strength is observed due to the diminishing micro-aggregate effect. The optimal balance between the filling effect and the micro-aggregate effect is achieved at a quicklime content of 25 wt.%, resulting in a peak cylinder compressive strength of 13 MPa.
(2) Despite the augmentation in hydration product content with increasing quicklime addition, which positively impacts the filling effect, the resultant surge in water consumption during the granulation process leads to a reduction in initial defects within the FAAs. This phenomenon, in turn, results in a decrease in apparent density and a progressive increase in water absorption.
(3) The water absorption capacity of the FAAs, measured after 1 h, falls within the range of 14.1% to 23.98%, while the 24 h absorption varies between 20.58% and 26.37%. The apparent density, ranging from 1430 kg/m^3^ to 1516 kg/m^3^, aligns with the lightweight material criteria. Furthermore, when the quicklime content is within the range of 10 wt.% to 45 wt.%, the FAAs satisfy the specifications for high-strength lightweight aggregates, as outlined in the national standards.

## Figures and Tables

**Table 1:** Main chemical composition of fly ash (FA) and cement (wt.%).

Chemical Composition	Fly Ash (%)	Cement (%)
SiO₂	49.7	21.3
Al₂O₃	32	5.1
Fe₂O₃	5.6	3.2
CaO	7.2	63.5
MgO	2.1	3.1
K₂O	1.4	1.2
Na₂O	0.3	0.4
SO₃	0.2	1.3
LOI	1.5	0.9

Note: LOI—loss of ignition.

**Table 2:** Mix proportions of FAAs (wt.%).

Notation	Core (%)		Shell (%)		Water (%)
	Quicklime	Fly Ash	Quicklime	Fly Ash	
L5F95	5	95	15	85	35.15
L10F90	10	90	15	85	41.80
L15F85	15	85	15	85	42.55
L20F80	20	80	15	85	45.55
L25F75	25	75	15	85	48.15
L30F70	30	70	15	85	52.05
L35F65	35	65	15	85	57.75
L40F60	40	60	15	85	58.15
L45F95	45	55	15	85	68.40

Note: The shell/core ratio is 0.04 for all samples; water—the total water consumption during the granulation process, calculated as the sum of m_1_ and m_2_ divided by m.

**Table 3:** CaO/SiO_2_ ratio of raw materials and hydration products.

Notation	CaO/SiO_2_ ^a^	R (wt.%)	CaO/SiO_2_ ^b^
L5F95	0.15	70.94	0.53
L10F90	0.26	63.83	0.72
L15F85	0.38	48.71	0.74
L20F80	0.51	45.72	0.94
L25F75	0.66	39.48	1.09
L30F70	0.83	30.22	1.19
L35F65	1.03	25.57	1.38
L40F60	1.26	17.94	1.54
L45F55	1.53	14.96	1.80

Note: CaO/SiO_2_ ^a^—Ca/Si ratio of the raw materials; CaO/SiO_2_ ^b^—Ca/Si ratio of the hydration products; R-insoluble matter.

## Data Availability

The original contributions presented in this study are included in the article. Further inquiries can be directed to the corresponding authors.
